# Post-Mortem Examinations on Hospital Patients

**Published:** 1897-12-25

**Authors:** 


### POST-MORTEM EXAMINATIONS ON HOSPITAL PATIENTS.

A subject which is a constant cause of trouble to hospital authorities, and often gives rise to angry discussion and hostile criticism in the Press, is the question of the right of hospital managers to permit post-mortem examinations to be made on such patients as die within their walls. We believe that legally, in default of special testamentary disposition, although the hospital authorities have no statutory right to examine the body, no one has any greater right to prevent them from so doing, and that if a post-mortem examination were once made the police would not institute any proceedings, and that any private individual who might choose to intervene would find it far from easy to establish any claim for damages or to impose any penalty on those who had done the deed. With hospitals, however, the matter is not entirely one of law. The primary object of hospitals being the cure of the sick, it is, to say the least of it, important that the sick should not be deterred from entering their doors, and with this object it is of great importance that they should, as the saying is, "stand well with the public." One of the great safeguards which the public possesses in regard to all hospitals which are conducted on the voluntary system which obtains in England, is that the hospitals depend for their support upon subscriptions, and that the subscriptions, the donations, and the legacies, by which they are supported, depend entirely upon their popularity, their freedom from reproach, and the absence of any "scandals" in regard to their management. Hence, the managers of our great hospitals are always unwilling to adopt any course which can be twisted into the semblance of an oppression or a scandal, even by the wit of a reporter for an evening paper. It may then be asked, Why do not hospital managers at once abolish the post-mortems altogether? To this the answer is plain. The object of the hospitals is not merely to give to their patients the first and handiest medicine which comes in the way, but to give them the best advice and the most scientific treatment possible, and if this is to be done the best and the most scientific men must be attracted to their staffs. If hospitals are to continue to offer to their patients the best possible treatment, they must offer to their medical officers the best possible opportunity for the prosecution of scientific researches as to the nature of the diseases which come under their care. The whole matter, like so many other matters in England, becomes one of compromise. Custom takes the place of right, and in practice it is found that what is customary is not difficult to carry out, however ill-defined it may be in law. With the object of finding out what is the general custom in regard to this matter, we have made inquiries of the secretaries of the chief London hospitals, and have elicited a good deal of information which we think will be of interest to hospital managers throughout the country. The replies which we have received have been tabulated in the following form:—

| QUESTION. | NAME OF INSTITUTION. | | | | | | | | | | |
|---|---|---|---|---|---|---|---|---|---|---|---|
| | St. Mary's. | St. George's. | Guy's. | St. Bart's. | London. | Middlesex. | Westminster. | King's College. | Univ. College. | Charing Cross. | Consumption (Brompton). |
| Is it the rule to hold a post-mortem upon every person dying in hospital? | No. | — | Yes. | — | By no means. | Yes; unless friends have previously objected. | Yes. | No. | — | Yes. | Yes; if possible. |
| Is the post-mortem made without reference to the friends? | No. | No. | Yes. | No. | Reference is always made to the friends. | Yes. | No. | No. | 24 hours are allowed to elapse between death and the P.-M. | Yes. | Never. |
| Is it delayed until friends have been communicated with, and permission obtained? | Always. | Yes. | No; but time is generally allowed for objection to be made. | Yes. | The consent of the friends is first obtained. | No. | Yes. | Yes. | — | It is the rule to make it 12 hours after death unless friends object. | Yes. |
| Are post-mortems ever held where the patients' friends refuse? | No. | No; except upon order of Coroner. | If serious objection is made a post-mortem is not held. | No; unless under order of Coroner. | A Coroner's order for a post-mortem overrides a friend's refusal. | No. | No. | No. | No. | No. | No. |

We would also draw attention to the following notice, which is fixed in a conspicuous place in the office at Guy's Hospital, where tickets of admission are given to patients and their friends :—

"Notice to Friends and Relatives of Deceased Patients.

" Extract from the minutes of the Court of Committees of Governors of Guy's Hospital, held September 7th, 1870: 'The Governors reserve to themselves, in the interest of the public, and as one of the conditions of admission to the hospital, the right of causing a post-mortem examination to be made of the body of every patient who dies within the hospital by the pathologist or his representative, for the purpose of accurately determining the causes of death. In the event of the friends or nearest relatives being opposed to such an examination they are to communicate their wishes to the superintendent, who will submit their objections to the medical officer who had charge of the deceased patient, and if he is of opinion that there is no urgent need for a post-mortem examination the superintendent is authorised to dispense with it.'—E. C. Perry, Superintendent, Guy's Hospital, 12th October, 1870."

